# A Novel De Novo GATA Binding Protein 3 Mutation in a Turkish Boy with Hypoparathyroidism, Deafness, and Renal Dysplasia Syndrome

**DOI:** 10.4274/jcrpe.2249

**Published:** 2015-12-03

**Authors:** Gül Yeşiltepe Mutlu, Heves Kırmızıbekmez, Akie Nakamura, Maki Fukami, Şükrü Hatun

**Affiliations:** 1 Zeynep Kamil Gynecologic and Pediatric Training and Research Hospital, Clinic of Pediatric Endocrinology, İstanbul, Turkey; 2 National Research Institute for Child Health and Development, Setagaya, Japan; 3 Kocaeli University Faculty of Medicine, Department of Pediatric Endocrinology and Diabetes, Kocaeli, Turkey

**Keywords:** GATA binding protein 3 gene, hypoparathyroidism, deafness, renal dysplasia

## Abstract

Hypoparathyroidism, deafness, and renal dysplasia (HDR; OMIM 146255) syndrome is a rare disease, inherited dominantly and found to be related with GATA3 (GATA binding protein 3) gene mutations. A 13-year and 8-month-old boy who presented with hypocalcemia was diagnosed with hypoparathyroidism. He also had dysmorphic facial features, renal anomaly (pelvic kidney), and mild sensorineural hearing loss. His cranial computed tomography revealed multiple calcifications in bilateral centrum semiovale, corona radiata, and basal ganglions suggesting a persistent hypoparathyroidism. Thus, the presence of triad of HDR syndrome was considered, and genetic analysis using a next-generation sequencer identified a novel de novo missense mutation in exon 4 p.R276Q (c.827G>A) of GATA3 gene. This is the second patient who was reported to have a mutation in GATA3 gene from Turkey. In conclusion, although HDR syndrome is a rare condition, it should be kept in mind in patients with hypoparathyroidism. Classical triad can easily be identified if patients diagnosed with hypoparathyroidism are also evaluated with a urinary tract ultrasound and an audiometer.

**WHAT IS ALREADY KNOWN ON THIS TOPIC?**

Hypoparathyroidism, deafness, and renal dysplasia is a rare syndrome, generally inherited dominantly and characterized with classic triad of hypoparathyroidism, deafness, and renal dysplasia. It is usually related to GATA3 (GATA binding protein 3) gene mutations.

**WHAT THIS STUDY ADDS?**

A novel heterozygous missense mutation p.R276Q(c.827G>A) in exon 4 of GATA3 gene was identified by DNA sequencing. The lack of this mutation in family members suggests that this is a de novo mutation, however, presence of renal anomaly in the mother suggested plausible mosaic somatic mutation in her ovaries and kidneys.

## INTRODUCTION

Hypoparathyroidism, deafness, and renal dysplasia (HDR; OMIM 146255) syndrome is a rare disease which is first defined by Barakat et al (1) in 1977. It is characterized by hypoparathyroidism, sensorineural deafness, and renal dysplasia, inherited dominantly, and is found to be related with GATA3 (GATA binding protein 3) gene mutations. This gene is located on 10p15 and is essential in the embryonic development of the parathyroid glands, auditory system, and kidneys (2). DiGeorge syndrome (DGS) is also characterized by hypoparathyroidism. The locus related with DGS 2 is along with GATA3, and deletions of distal 10 p region lead to phenotypic findings of DGS in addition to HDR syndrome (3).

A patient clinically diagnosed with HDR syndrome having a novel mutation on the GATA3 gene is presented in this case report.

## CASE REPORT

A 13-year and 8-month-old boy was admitted because of spasm of the hands for approximately two weeks. Hypocalcemia was detected, and the patient was referred to pediatric endocrinology clinic. He was a full-term infant born from non-consanguineous parents with a birth weight of 2,400 gr. Neuromotor development was delayed and school performance was poor. He did not have a history of chronic disease, medication, or surgery. Family history did not reveal mental retardation, hypocalcemia, renal disease, and deafness.

**Figure 1:**
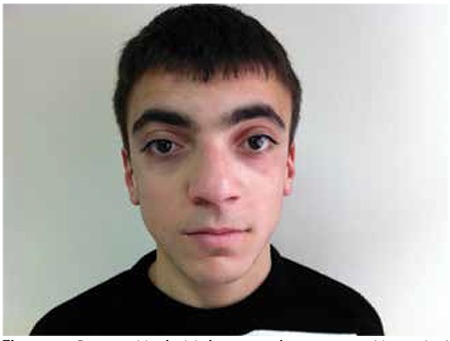
Dysmorphic facial features of our case with marked hypertelorism

He appeared well and conscious in physical examination. The heart rate was 94/min, the blood pressure was 120/75 mmHg, and Chvostek sign was present. His height was 165.9 cm (50-75 p), weight was 57.5 kg (50-75 p). He had facial dysmorphism characterized by hypertelorism (Figure 1), low-set ears, and highly-arched palate. Thyroid gland was non-palpable, and pubertal stage was Tanner stage 5. Cataract was not detected in ophthalmologic examination. Laboratory findings were as follows: serum calcium (Ca) was 6.7 mg/dL, phosphorus (P): 9.5 mg/dL, parathyroid hormone (PTH): 20 pg/mL, 25 hydroxy-vitamin D (25OHD): 27 ng/mL, magnesium: 1.8 mg/dL, Ca/creatinin ratio in spot urine: 0.05. Serum albumin, glucose, electrolytes, renal functions, and complete blood count were normal. Early morning cortisol was 14.7 mcg/dL, thyroid functions were normal, and thyroid antibodies were negative.

**Figure 2:**
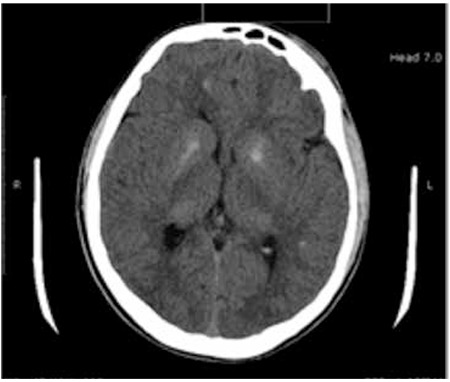
Multiple calcifications in bilateral centrum semiovale, corona radiata and basal ganglions were shown by cranial computed tomography

The patient was diagnosed to have hypoparathyroidism. Oral calcitriol and Ca replacement treatment was commenced. Renal ultrasonography (USG) revealed that the right kidney was 96x57 mm in normal localization, while the left kidney was ectopic and significantly smaller than the right one. Decreased renal function in the ectopic kidney was detected by dimercaptosuccinic acid (DMSA) scan. Multiple calcifications in bilateral centrum semiovale, corona radiate, and basal ganglions were shown by cranial computed tomography (CT) (Figure 2), echocardiography was normal, and audiometer revealed bilateral mild sensorineural hearing loss. Intelligence quotient (IQ) score was reported as 57 in Weschler Intelligence Scale For Children test, suggesting mild mental retardation.

Clinical and laboratory findings were supporting the diagnosis of HDR syndrome. Family members were also evaluated in terms of hypocalcemia, hearing loss, and renal dysgenesis. His mother had horse-shoe kidney, but others did not have any of the suspected findings.

**Figure 3:**
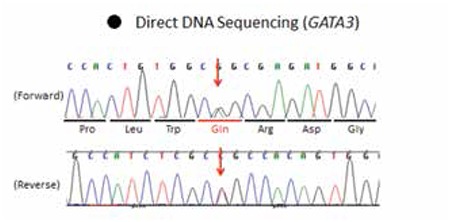
A heterozygous novel missense mutation (R276Q) (c.824G>A) was identified in exon 4 of GATA3 gene

The karyotype of this patient was 46, XY. Genetic analysis using a next-generation sequencer identified a missense mutation in exon 4 p.R276Q (c.827G>A) of GATA3 gene (Figure 3). This mutation was confirmed by Sanger direct sequencing. The mutation was not detected in his parents, brother, or sister.

## DISCUSSION

Since HDR syndrome (Barakat syndrome) is a very rare disorder, its prevalence is not known exactly. A limited number of patients were reported in the literature (4).

Nearly 90% of patients with HDR syndrome have hypoparathyroidism and hearing loss, while 80% have renal dysplasia. Occurrence of findings is variable in reported cases. Some of the patients present with neuromuscular irritability and convulsions (5), while some of them are asymptomatic for many years despite severe hypocalcemia (6). This patient had symptomatic hypocalcemia for two weeks, however, the presence of cerebral calcifications on CT suggested a longer time of disease duration in our case. Although the etiopathogenesis of intracranial calcifications is not well-defined, calcifications are thought to be due to hyperphosphatemia rather than hypoparathyroidism itself. Interestingly, ectopic calcifications due to hyperphosphatemia are located on the vascular structure if it is caused by renal failure, while they are located in brain tissue if caused by hypoparathyroidism (7). Intracranial calcifications associated with HDR have been previously reported in the literature (6,8).

Renal disorders, which are the most heterogeneous component of this syndrome, include either functional or structural abnormalities. Renal hypoplasia, dysplasia, cystic kidneys, vesicoureteric reflux, nephritic syndrome, pelvicalyceal abnormality, hematuria, proteinuria, proximal and distal renal tubular acidosis with nephrocalcinosis were reported in association with HDR. Our patient had a pelvic kidney which has been previously reported in one patient (9).

Mental disabilities in the HDR syndrome are not common. Neuropsychiatric symptoms are thought to be related with calcifications of basal ganglia. The neurologic examination of our patient was completely normal despite the intracranial calcifications. Gaynor et al (9) similarly reported one case with HDR having motor and mental retardation.

No typical facial appearance has been described for this syndrome. Hypertelorism was the most prominent dysmorphic finding in our patient. There are few HDR cases with hypertelorism which were reported in the literature. These cases were found to have deletion in the chromosome 10 (3,10,26). Despite having only a single nucleotide change, our patient interestingly have dysmorphic appearance similar to these cases. This condition does not support a genotype-phenotype correlation in HDR syndrome. The phenotypical features of the cases with HDR syndrome reported in the literature are summarized in Table 1.

A heterozygous missense mutation p.R276Q (c.827G>A) in exon 4 of GATA3 gene was identified by DNA sequencing. This mutation resided within the first zinc finger domain. This mutation has not been identified in patients with HDR syndrome, however, Nesbit et al (20) previously performed in vitro analysis of artificially generated p.R276Q mutant and found that this mutant could not stabilize DNA binding. The lack of this mutation in family members of the present case suggests that this is a de novo mutation, although HDR syndrome cases are generally reported to be inherited dominantly (3). The GATA3 mutation was absent in the proband’s mother with horse-shoe kidney. Our results indicate that the kidney malformation in the mother is unrelated to the GATA3 mutation. However, we cannot exclude the possibility that the mother has a mosaic somatic GATA3 mutation in the kidney and ovary, but not in peripheral lymphocytes. Although somatic mosaicism of GATA3 mutations has not been reported so far, such phenomenon was reported in several congenital diseases such as osteogenesis imperfecta type 2 (27). There are also some examples of somatic mosaic mutations in PIK3CA and FMR1 genes that were only detected in relevant tissues but not in the blood (28,29). Unfortunately, we could not perform the GATA3 gene mutation analysis in the peripheral tissues of the parents.

This is the second case with HDR syndrome and GATA3 mutation that was reported from Turkey. The first patient was a 4-month-old infant who had a previously known mutation (p.R367X) in GATA3 gene (5).

In conclusion, although HDR syndrome is a rare condition, it should be in patients with hypoparathyroidism. Classical triad can easily be identified if patients diagnosed with hypoparathyroidism are also evaluated with a urinary tract USG and an audiometer.

## Figures and Tables

**Table 1:**
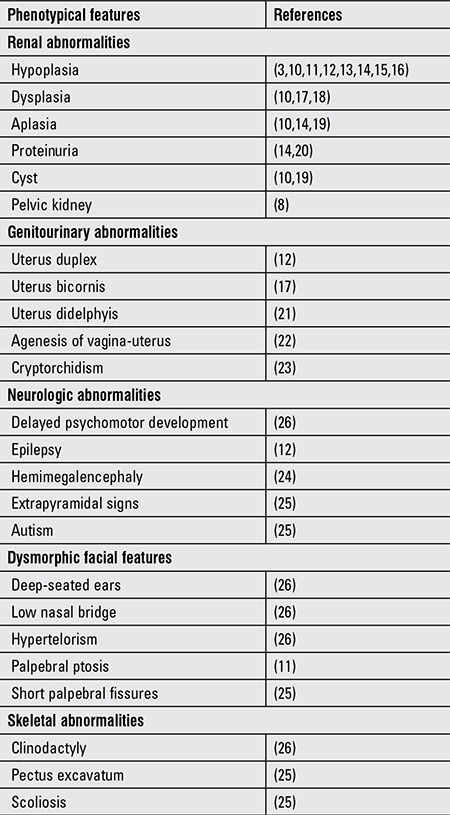
The phenotypical features which were detected in the cases with hypoparathyroidism, deafness, and renal dysplasia syndrome

## References

[1] Barakat AY, D’Albora JB, Martin MM, Jose PA (1977). Familial nephrosis, nerve deafness, and hypoparathyroidism. J Pediatr.

[2] Van Esch H, Devriendt K (2001). Transcription factor GATA3 and the human HDR syndrome. Cell Mol Life Sci.

[3] Fukami M, Muroya K, Miyake T, Iso M, Kato F, Yokoi H, Suzuki Y, Tsubouchi K, Nakagomi Y, Kikuchi N, Horikawa R, Ogata T (2011). GATA3 abnormalities in six patients with HDR syndrome. Endocr J.

[4] Maleki N, Bashardoust B, IranparvarAlamdari M, Tavosi Z (2013). Seizure, deafness, and renal failure: a case of barakat syndrome. Case Rep Nephrol.

[5] Döneray H, Usui T, Kaya A, Donmez AS (2015). The first turkish case with hypoparathyroidism, deafness and renal dysplasia (HDR) syndrome. J Clin Res Pediatr Endocrinol.

[6] Nanba K, Usui T, Nakamura M, Toyota Y, Hirota K, Tamanaha T, Kawashima ST, Nakao K, Yuno A, Tagami T, Naruse M, Shimatsu A (2013). A novel GATA3 nonsense mutation in a newly diagnosed adult patient of hypoparathyroidism, deafness, and renal dysplasia (HDR) syndrome. Endocr Pract.

[7] Fujita T (2004). Mechanism of intracerebral calcification in hypoparathyroidism. Clin Calcium.

[8] Rizvi I, Ansari NA, Beg M, Shamim MD (2012). Widespread intracranial calcification, seizures and extrapyramidal manifestations in a case of hypoparathyroidism. N Am J Med Sci.

[9] Gaynor KU, Grigorieva IV, Nesbit MA, Cranston T, Gomes T, Gortner L, Thakker RV (2009). A missense GATA3 mutation, Thr272Ile, causes the hypoparathyroidism, deafness, and renal dysplasia syndrome. J Clin Endocrinol Metab.

[10] Lichtner P, König R, Hasegawa T, Van Esch H, Meitinger T, Schuffenhauer S (2000). An HDR (hypoparathyroidism, deafness, renal dysplasia) syndrome locus maps distal to DiGeorge syndrome region on 10p13/14. J Med Genet.

[11] Ali A, Christie PT, Grigorieva IV, Harding B, Van Esch H, Ahmed SF, Bitner-Glindzicz M, Blind E, Bloch C, Christin P, Clayton P, Gecz J, Gilbert-Dussardier B, Guillen-Navarro E, Hackett A, Halac I, Hendy GN, Lalloo F, Mache CJ, Mughal Z, Ong AC, Rinat C, Shaw N, Smithson SF, Tolmie J, Weill J, Nesbit MA, Thakker RV (2007). Functional characterization of GATA3 mutations causing the hypoparathyroidism-deafness-renal (HDR) dysplasia syndrome: insight into mechanisms of DNA binding by the GATA3 transcription factor. Hum Mol Genet.

[12] Ferraris S, Del Monaco AG, Garelli E, Carando A, De Vito B, Pappi P, Lala R, Ponzone A (2009). HDR syndrome: a novel “de novo” mutation in GATA3 gene. Am J Med Genet A.

[13] Nakamura A, Fujiwara F, Hasegawa Y, Ishizu K, Mabe A, Nakagawa H, Nagasaki K, Jo W, Tajima T (2011). Molecular analysis of the GATA3 gene in five Japanese patients with HDR syndrome. Endocr J.

[14] Labastie MC, Bories D, Chabret C, Gregoire JM, Chretien S, Romeo PH (1994). Structure and expression of the human GATA3 gene. Genomics.

[15] Muroya K, Hasegawa T, Ito Y, Nagai T, Isotani H, Iwata Y, Yamamoto K, Fujimoto S, Seishu S, Fukushima Y, Hasegawa Y, Ogata T (2001). GATA3 abnormalities and the phenotypic spectrum of HDR syndrome. J Med Genet.

[16] Al-Shibli A, Al Attrach I, Willems PJ (2011). Novel DNA mutation in the GATA3 gene in an Emirati boy with HDR syndrome and hypomagnesemia. Pediatr Nephrol.

[17] Ohta M, Eguchi-Ishimae M, Ohshima M, Iwabuki H, Takemoto K, Murao K, Chisaka T, Yamamoto E, Higaki T, Isoyama K, Eguchi M, Ishii E (2011). Novel dominant-negative mutant of GATA3 in HDR syndrome. J Mol Med (Berl).

[18] Van Esch H, Groenen P, Nesbit MA, Schuffenhauer S, Lichtner P, Vanderlinden G, Harding B, Beetz R, Bilous RW, Holdaway I, Shaw NJ, Fryns JP, Thakker RV, Devriendt K (2000). GATA3 haplo-insufficiency causes human HDR syndrome. Nature.

[19] Kobayashi H, Kasahara M, Hino M, Yoshimura H, Takahara S, Ikeda K, Son C, Iwakura T, Yoshimoto A, Ishihara T, Ogawa Y (2006). A novel heterozygous deletion frameshift mutation of GATA3 in a Japanese kindred with the hypoparathyroidism, deafness and renal dysplasia syndrome. J Endocrinol Invest.

[20] Nesbit MA, Bowl MR, Harding B, Ali A, Ayala A, Crowe C, Dobbie A, Hampson G, Holdaway I, Levine MA, McWilliams R, Rigden S, Sampson J, Williams AJ, Thakker RV (2004). Characterization of GATA3 mutations in the hypoparathyroidism, deafness, and renal dysplasia (HDR) syndrome. J Biol Chem.

[21] Sun Y, Xia W, Xing X, Li M, Wang O, Jiang Y, Pei Y, Ye P, Liu H, Hu Y, Meng X, Zhou X (2009). Germinal mosaicism of GATA3 in a family with HDR syndrome. Am J Med Genet A.

[22] Hernandez AM, Villamar M, Rosello L, Moreno-Pelayo MA, Moreno F, Del Castillo I (2007). Novel mutation in the gene encoding the GATA3 transcription factor in a Spanish familial case of hypoparathyroidism, deafness, and renal dysplasia (HDR) syndrome with female genital tract malformations. Am J Med Genet A.

[23] Moldovan O, Carvalho R, Jorge Z, Medeira A (2011). A new case of HDR syndrome with severe female genital tract malformation: comment on “Novel mutation in the gene encoding the GATA3 transcription factor in a Spanish familial case of hypoparathyroidism, deafness, and renal dysplasia (HDR) syndrome with female genital tract malformations” by Hernández et al. Am J Med Genet A.

[24] Melis D, Genesio R, Boemio P, Del Giudice E, Cappuccio G, Mormile A, Ronga V, Conti A, Imperati F, Nitsch L, Andria G (2012). Clinical description of a patient carrying the smallest reported deletion involving 10p14 region. Am J Med Genet A.

[25] Adachi M, Tachibana K, Asakura Y, Tsuchiya T (2006). A novel mutation in the GATA3 gene in a family with HDR syndrome (Hypoparathyroidism, sensorineural Deafness and Renal anomaly syndrome). J Pediatr Endocrinol Metab.

[26] Verri A, Maraschio P, Devriendt K, Uggetti C, Spadoni E, Haeusler E, Federico A (2004). Chromosome 10p deletion in a patient with hypoparathyroidism, severe mental retardation, autism and basal ganglia calcifications. Ann Genet.

[27] Wallis GA, Starman BJ, Zinn AB, Byers PH (1990). Variable expression of osteogenesis imperfecta in a nuclear family is explained by somatic mosaicism for a lethal point mutation in the alpha 1 (I) gene (COL1A1) of type 1 collagen in a parent. Am J Hum Genet.

[28] Cohen AS, Townsend KN, Xiang QS, Attariwala R, Borchers C, Senger C, Picker W, Levi J, Yewchuk L, Tan J, Eydoux P, Lum A, Yong SL, McKinnon ML, Lear SA, Everett R, Jones SJ, Yip S, Gibson WT (2014). Somatic mosaicism for the p.His1047Arg mutation in PIK3CA in a girl with mesenteric lipomatosis. Am J Med Genet A.

[29] Luo S, Huang W, Xia Q, Xia Y, Du Q, Wu L, Duan R (2014). Cryptic FMR1 mosaic deletion in a phenotypically normal mother of a boy with fragile X syndrome: case report. BMC Med Genet.

